# EIF1AX c.338-2A>T splice site mutation in a patient with trabecular adenoma and cytological indeterminate lesion

**DOI:** 10.20945/2359-3997000000208

**Published:** 2020-03-18

**Authors:** Maria Grazia Castagna, Tania Pilli, Fabio Maino, Carlotta Marzocchi, Giovanni Di Cairano, Silvia Cantara

**Affiliations:** 1 Department of Medical, Surgical and Neurological Sciences University of Siena Siena Italy Department of Medical, Surgical and Neurological Sciences, University of Siena, Siena, Italy

## Abstract

The *EIF1AX* gene mutations have been recently associated with papillary thyroid carcinoma and anaplastic thyroid cancer. According with these reports, the gene as been considered as a drive gene for thyroid cancer development. However, the occurrence of these alterations in benign thyroid lesions is not known and is still under investigation. Some authors have already reported the presence of *EIF1AX* variants in follicular adenomas and hyperplastic nodules. Here, we describe for the first time a case of a man with the *EIF1AX c.338-2A>T* splice site mutation in an indeterminate FNA lesion with trabecular adenoma at final histology in the absence of other pathogenetic mutations, demonstrating that further studies are required to better understand *EIF1AX* role in the tumorigenesis of thyroid carcinoma.

## INTRODUCTION

Around 15-30% of all thyroid lesions investigated by fine needle aspiration cytology (FNAC) are of indeterminate origin. Among these, 5-15% are atypia of undetermined significance/follicular lesion of undetermined significance (AUS/FLUS) and 20-30% are follicular neoplasm/suspicious for follicular neoplasm (FN) (Bethesda Classification System) (1). The overall risk of cancer is 34-42%% (1) and thus to apply surgery to all indeterminate lesions will end in approximately 60-70% of unnecessary operations. The identification of genetic alterations specific for differentiated thyroid cancer have supplied molecular markers to be searched for in the material obtained by fine needle aspiration (FNA), thus enhancing the diagnostic accuracy of traditional cytology. The European Thyroid Association (ETA) recently published a document (2) about clinical recommendations on the use of molecular diagnostics in the evaluation of indeterminate cytologies. In particular, ETA guidelines recommend the search of at least BRAF, RAS point mutations (N-, H-, and K-RAS) and RET/PTC1-3, PAX8/PPARG rearrangements (7 gene panel). This panel has been shown in several reports to have a good sensitivity and specificity with a negative predictive value of 56-100% (2-7). With the increased of molecular techniques and the advent of next generation sequencing a larger mutation panel was proposed for the molecular diagnosis of indeterminate lesions (8-15). The Thyroseq platform has been shown a sensitivity of 74-91%, specificity 50-92% and a NPV of 89-97% (10,11,13,14,16-24). In addition to the 7 gene panel, Thyroseq suggest the search of several variants such as ALK, NTRK3 gene fusions and mutations of hTERT, EIF1AX that considered together account for 12% of all thyroid cancers.

## CASE REPORT

**Figure 1:**
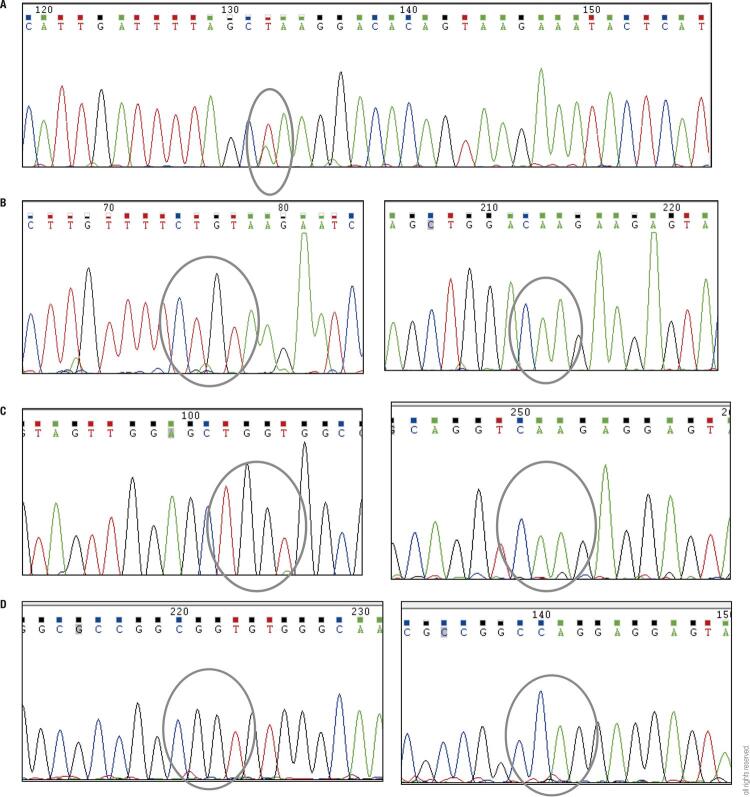
(A) Electropherogram for EIF1AX exon 6. The splice site region is evidenced in the circle. (B) Sequences for NRAS codon 12 (left) and codon 61 (right). (C) Sequences for KRAS codon 12 (left) and codon 61 (right). D: Sequences for HRAS codon 12 (left) and codon 61 (right). For all, in the circle is evidences the hot spot site.

A 55 year old man was referred to our clinic of Endocrinology, University-Hospital of Siena because of the presence of multinodular goiter. Blood samples showed normal levels of thyroperoxidase (AbTPO) and thyroglobulin antibodies. The patient was euthyroid with 0.5 µU/ml of thyroid-stimulating hormone (TSH) (range 0.4-4.0 µU/ml), 3.6 pg/ml of free triiodothyronine (FT3) (range 2.5-4.5 pg/mL), and 7.8 pg/mL of free thyroxine (FT4) (range 5.8-16.40 pg/mL). At physical examination, a palpable nodule of ~1.5 cm in size was detected in the left lobe of the thyroid gland. The neck ultrasound confirmed the presence of a dominant thyroid nodule with sonographic features of solid hypoechoic, lobulated margins, taller than wider (2.0x1.6x1.7 cm) and intranodular vascularity. Therefore, a FNAC was performed under ultrasound guidance and cytological results revealed a AUS/FLUS lesion. According with ETA guidelines, the genetic analysis was applied to the lesion in order to refine pre-operative diagnosis. We first verified thyroid origin of the FNA material by analyzing TPO and TSHR expression. Then, the FNA specimen was sequenced for the seven gene panel. In addition, EIF1AX (exons 2, 5 and 6), ALK fusions, hTERT and p53 (exons 5 and 7) point mutations were analyzed. The FNA sample was negative for all (Table 1), but was found to carry a c.338-2A>T splice site mutation (Figure 1A) in the eukaryotic translation initiation factor 1A, X-linked (EIF1AX) which abolish the splice acceptor site of exon 6 resulting in two alternative spliced mRNA (25). Given the association of *EIF1AX* as a driver gene in papillary thyroid carcinoma (PTC) and the presence is some cases of anaplastic thyroid cancer (ATC) (26), the patient was recommended to undergo thyroid surgery. The final pathologic diagnosis was trabecular adenoma (a benign lesion characterized by cells which are closely packed to form cords with only a few small follicles) with presence of Hürthle cells. Genomic analysis of the surgical specimen confirmed the presence of the a c.338-2A>T nucleotide change in *EIF1AX* in the absence of the other mutations (BRAF, N-, H-, and K-RAS, RET/PTC1-3, PAX8/PPARG, ALK, hTERT and p53).

**Table 1:** Panel analyzed and results of sequencing for our patient

Oncogene analyzed	Results for mutation
BRAF V600E	Negative
BRAF K601E	Negative
RET/PTC1 and RET/PTC3	Negative
PAX8/PPARg	Negative
hTERT (C228T, C25OT)	Negative
ALK fusions	Negative
H-N-K RAS (condons 12 and 61)	Negative
TP53 (exons 5 and 7)	Negative
EIF1AX (exons 2, 5 and 6)	c.338-2A>T splice site

### Comment

The Eukaryotic Translation Initiation Factor 1A, X-Linked (*EIF1AX*) gene encodes a crucial eukaryotic translation initiation factor. The protein is required for the binding of the 43S complex (a 40S subunit, eIF2/GTP/Met-tRNAi and eIF3) to the 5’ end of capped RNA (GeneCard). Furthermore, over-expression of E1F1AX increases expression of Cyclin D1 inducing cell proliferation. Recently, variations in this gene have been associated with thyroid cancer both papillary and anaplastic (26). Mostly, these reports have identified the c.338-1G>T splice site mutation in combination with RAS mutations (mainly NRAS p.Q61K and p.Q61R) (27-28). Our patient was negative for RAS mutations (figure 1B, C and D). The prevalence of EIF1AX in benign lesions is still under investigation. In 2016, Karunamurthy and cols. (27) found the A113_splice mutation in 7.4% of follicular adenomas and 1.3% of hyperplastic nodules in a series of 266 sample. Given the fact that the alteration was present also in 2.3% of PTC and 25% of ATC, they concluded that when found in thyroid FNA, EIF1AX confers approximately 20% risk of cancer. In the same year, Yoo and coworkers published a comprehensive analysis of the trascriptome and genome of follicular versus papillary thyroid cancer (29). They compared the RNA sequencing results of 77 PTCs, 48 follicular variant of PTC (FVPTC), 30 minimally invasive follicular thryoid cancer (miFTC) and 25 follicular adenomas (FA) to understand the differences in their molecular properties. EIF1AX was found only in 5.45% of the sample of miFTC or FA origin without the coexistence of BRAF or RAS mutations (29). Recently the c.338-1G>T was found for the first time in a case of Hurtle cell carcinoma (30). This patient carried no mutation in RAS genes but was found to have a mutation in the TP53 possibly responsible for the more aggressive behavior of the tumor (30). At contrast with this study, our patient was also negative for TP53 mutations (Table 1). Same group reported a rare case of a follicular adenoma harboring the C228T mutation in TERT gene, the Q61R mutation in HRAS, and the c.338-1G>T splice mutation in EIF1AX gene (31). In another study (32) in which 201 follicular-patterned thyroid tumors were analyzed, the authors found EIF1AX only in one case of adenoma concluding that, EIF1AX has limited impact on molecular diagnostics of thyroid tumors. Opposite conclusions are given by Jung and co-workers which state that EIF1AX together with EZH1, TSHR, and BRAF mutations may play roles in the initial development and progression of follicular tumors (33). A possible limitation of our study, could be the absence a scintigraphy to exclude the presence of a pre-toxic nodule. The exam was suggested to the patient, who refused it, preferring to undergo surgical treatment. Moreover, in pre-toxic nodule, TSHR mutations can be found even associated with EIF1AX mutations (33). Unfortunately, TSHR mutations were not evaluated in the present study, due to low material obtained from FNA (RNA: 12.4 ng/ul; DNA: 6.1 ng/ul) and from the post-surgical tissue specimen (RNA: 8 ng/ul and DNA: 4.3 ng/ul).

In conclusion, we describe for the first time the presence of EIF1AX c.338-2A>T splice site mutation in a trabecular adenoma with the absence of other known pathogenetic mutations and again we open the question whether *EIF1AX* splice region variants may represent an early genetic event in the molecular pathogenesis of follicular thyroid carcinoma or can be present in exclusively benign lesions without consequence on cell transformation. In this case, the aggressive phenotype described in other reports (27,28,30) can be associated only with the presence of RAS or TP53 variants.

All procedures followed were in accordance with the ethical standards of the responsible committee on human experimentation (institutional and national) and with the Helsinki Declaration of 1975, as revised in 2000. Informed consent was obtained from all patients for being included in the study.
